# Role of neuroimaging in multidisciplinary approach towards Non-Alzheimer’s dementia

**DOI:** 10.1007/s13244-015-0421-1

**Published:** 2015-07-24

**Authors:** Satya Narayana Patro, Rafael Glikstein, Prasad Hanagandi, Santanu Chakraborty

**Affiliations:** Division of Neuroradiology, The Ottawa Hospital, University of Ottawa, 1053 Carling Avenue, K1Y 4E9 Ottawa, ON Canada

**Keywords:** Dementia, Alzheimer, Atrophy, Myelinolysis, Ataxia

## Abstract

Dementia is defined as chronic deterioration of intellectual function and cognitive skills significant enough to interfere with the ability to perform daily activities. Recent advances in the treatment of dementia have renewed interest in the use of various neuroimaging techniques that can assist in the diagnosis and differentiation of various subtypes. Neuroimaging and computational techniques have helped the radiological community to monitor disease progression of various neurodegenerative conditions presenting with dementia, such as Alzheimer disease, frontotemporal lobe dementia (FTLD), progressive supranuclear palsy (PSP) and multisystem atrophy-cerebellar variant (MSA-C), and their response to newer therapies. Prompt identification of treatable or reversible forms of dementia, such as tumours, subdural haemorrhage and intracranial dAVF, is crucial for the effective management of these conditions. It is also prudent to recognize the imaging spectrum of metabolic, infective and autoimmune diseases with rapidly progressing dementia, such as methanol toxicity, central pontine myelinolysis (CPM), delayed post hypoxic leukoencephalopathy (DPHL), HIV, Creutzfeldt-Jakob Disease (CJD), Sjogren's syndrome, multiple sclerosis (MS), radiation necrosis and Fragile X-Associated Tremor/Ataxia Syndrome (FXTAS), which are difficult to treat and often require palliative care. This pictorial review emphasizes various non-Alzheimer’s dementia entities and discusses their imaging highlights.

*Teaching Points*

• *Non Alzheimer’s dementia constitutes a broad spectrum of conditions.*

• *Neuroimaging plays an important role in differentiating treatable from irreversible dementia.*

• *Neuroimaging is often non-specific in early stages of neurodegenerative conditions with dementia.*

• *Neuroimaging plays an important role in the multimodal approach towards management of dementia.*

## Introduction

Dementia is a disorder characterized by global impairment in cognition, social and occupational functioning severe enough to interfere with daily functioning and quality of life. Various studies have confirmed an exponential increase in dementia with age and a higher prevalence in older females [1]. Overall, dementia has a significant burdening impact onsocio-economic status and the health care system.

Alzheimer disease (AD) is the most common cause of primary dementia, constituting 60 % of all cases. Vascular dementia is the second most common form of dementia, which often coexists with AD. There are various other non-Alzheimer conditions, which may present with cognitive impairments and can be divided into the broad categories of neurodegenerative, inflammatory/infective, metabolic/genetic and miscellaneous. This pictorial review emphasizes the role of neuroimaging in evaluating various causes of non-Alzheimer’s dementia. We also highlight conditions with reversible dementia, where neuroimaging plays a significant role in management.

### Vascular dementia (VaD)

Binswanger and Alzheimer first described VaD and recognized the role of multiple infarctions and chronic ischemia in its etiopathogenesis. VaD is the most common cause of non-Alzheimer dementia in the aging population and often contributes to cognitive impairment in AD and other forms of so-called “mixed dementias” [2]. Unlike AD, there are no pathological criteria for the diagnosis of VaD. The clinical criteria are poorly understood and validated. It’s a heterogeneous syndrome rather than a distinct disorder, in which the underlying cause is cerebrovascular disease in some form and its ultimate manifestation is dementia [3]. Three common forms of ischemic lesions can result in VaD: 1) Large artery infarcts involving the cortex and subcortical regions due to thromboembolic occlusion of major intracranial arteries; 2) Small artery infarctions or lacunes attributed to arteriolosclerosis involving the penetrating arteries and affecting the thalamus, basal ganglia, internal capsule, brain stem and cerebellum; 3) Periventricular white matter disease resulting from chronic subcortical ischemia of small arteries affecting the neurons, oligodedrocytes and astrocytes. Multiple studies have shown that in nearly 6 to 32 % of cases with lobar and lacunar ischemic stroke, one can show post stroke cognitive impairment and dementia [4]. The presence of microbleeds, a manifestation of small vessel disease, is also associated with cognitive decline. The number of infarcts and anatomic distribution of ischemic stroke, rather than the volume, determines the development of VaD. The more vulnerable areas are the hippocampus, angular gyrus, cingulate gyrus, frontal lobe or deep gray and white matter (thalamus, fornix, basal forebrain, caudate, globus pallidus, and the genu or anterior limb of the internal capsule) [5]. Advanced age, increased severity of the stroke, recurrent strokes, white matter disease, cortical atrophy (particularly in the temporal lobe), hypertension, obesity, elevated homocysteine, hyperlipidemia, and diabetes mellitus are the common associated risk factors [6]. The clinical presentation varies depending on cortical or subcortical ischemic lesions. Cortical lesions tend to be sudden onset with more focal neurodeficits, whereas subcortical lacunar infarcts and white matter disease have more gradual or stepwise decline of cognitive functions.

**Fig. 1:**
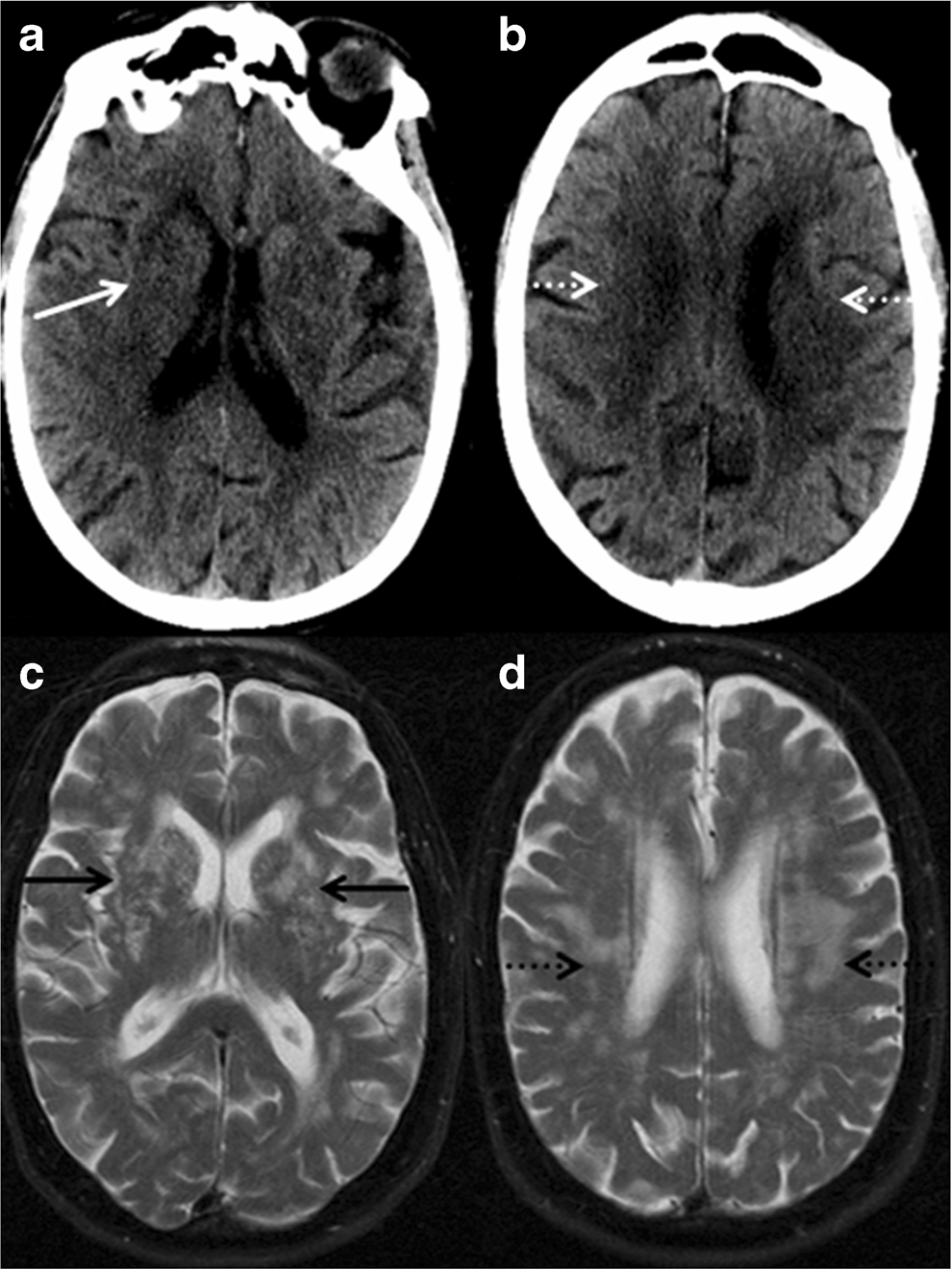
Vascular dementia. An 86-year-old man with multiple transient ischemic attacks (TIAs), ongoing changes in level of consciousness and memory. **a & b** noncontrast CT scan of the head shows multiple patchy confluent hypodensities in bilateral basal ganglia (*white arrow*), periventricular and subcortical white matter (*dashed white arrow*). **c & d** T2W MRI images of the brain revealing diffuse patchy and confluent hyperintensities in bilateral basal ganglia (*black arrows*) and white matter (*dashed black arrow*)

The diagnostic approach to patients with suspected VaD is similar to that of any other form of cognitive decline. Neuroimaging should be performed where there is history of stroke, vascular risk factors and clinical features atypical of AD. Magnetic resonance imaging (MRI) is considerably superior in sensitivity and specificity in detecting ischemic lesions compared to computed tomography (CT); however, radiological criteria are not adequate enough in differentiating between post-stroke patients with and without dementia [7]. Microangiopathic disease is the condition most commonly associated with vascular dementia and has non-specific imaging features. Confluent hypodensities in the periventricular and subcortical white matter (Fig. 1) with presence of cortical and lacunar infarcts are seen on CT. Likewise, MRI demonstrates confluent signal changes in the periventricular and subcortical white matter, deep grey nuclei and brain stem (Fig. 1). Increasing white matter lesion burden on serial MRI scans has been associated with accelerated cognitive decline in several studies [8]. Acute infarcts typically demonstrate T2W/FLAIR hyperintensity with diffusion restriction, whereas chronic infarcts exhibit volume loss with facilitated diffusion. GRE/SWI (Gradient Echo/Susceptibility Weighted Imaging) images are very sensitive in detecting microhaemorrhages noted in microangiopathic disease and other vascular conditions such as CADASIL (Cerebral Autosomal Dominant Arteriopathy with Subcortical Infarcts and Leukoencephalopathy), central nervous system (CNS) vasculitis and amyloid angiopathy. In summary, there are no uniform diagnostic criteria; however, a combination of clinical and neuropsychological assessment with neuroimaging inputs may help in the diagnosis and management of patients with VaD.

### Related conditions

#### CADASIL

**Fig. 2:**
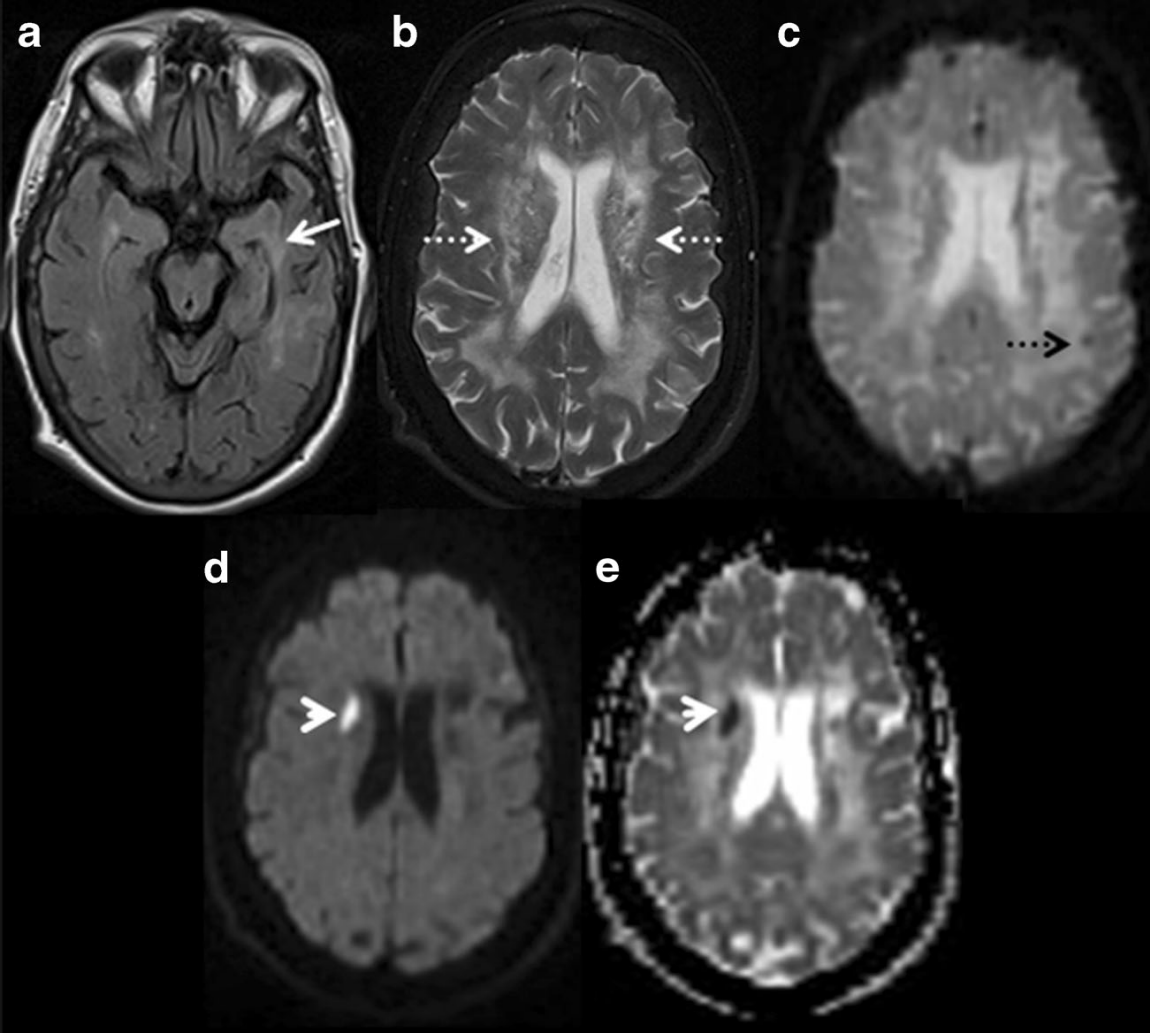
CADASIL. A 51-year-old female with left hand weakness, slurred speech and dysarthria with progressive cognitive decline. **a & b** axial FLAIR and T2W images of brain demonstrate patchy confluent hyperintensities in periventricular (*white dashed arrows*) and subcortical white matter with typical involvement of temporal lobes (*white arrow*). **c** axial GRE image shows presence of microhaemorrhage in the subcortical white matter (black dotted arrow). **d & e** axial DWI and ADC map of brain reveal focal area of restriction of diffusion (*white arrow head*) in the right corona radiata, suggesting acute infarct

CADASIL is an inherited vasculopathy with subcortical dementia of Binswanger type. It affects the younger population, beginning in the fourth to fifth decade, and commonly presents with recurrent transient ischemic attacks, strokes, migraine and cognitive decline [9]. The etiopathogenesis is characteristic vasculopathy of small and medium size arteries. MRI is the most useful neuroimaging technique to demonstrate radiological picture of CADASIL. Symmetric and confluent T2W/FLAIR hyperintensities in periventricular and subcortical white matter are commonly demonstrated in CADASIL. Anterior temporal lobe white matter involvement is noted in nearly 90 % of cases and uncommon in other microvascular diseases. Corpus callosum and insular T2W hyperintensities are also characteristic findings. DWI and GRE/SWI sequences are basically helpful in demonstrating occasional areas of associated acute infarcts and microhaemorrhages (Fig. 2) [10]. Brain atrophy is another important feature of CADASIL, likely resulting from subcortical ischemia and strongly correlated with cognitive decline.

#### Chronic subdural hematoma (SDH)

**Fig. 3:**
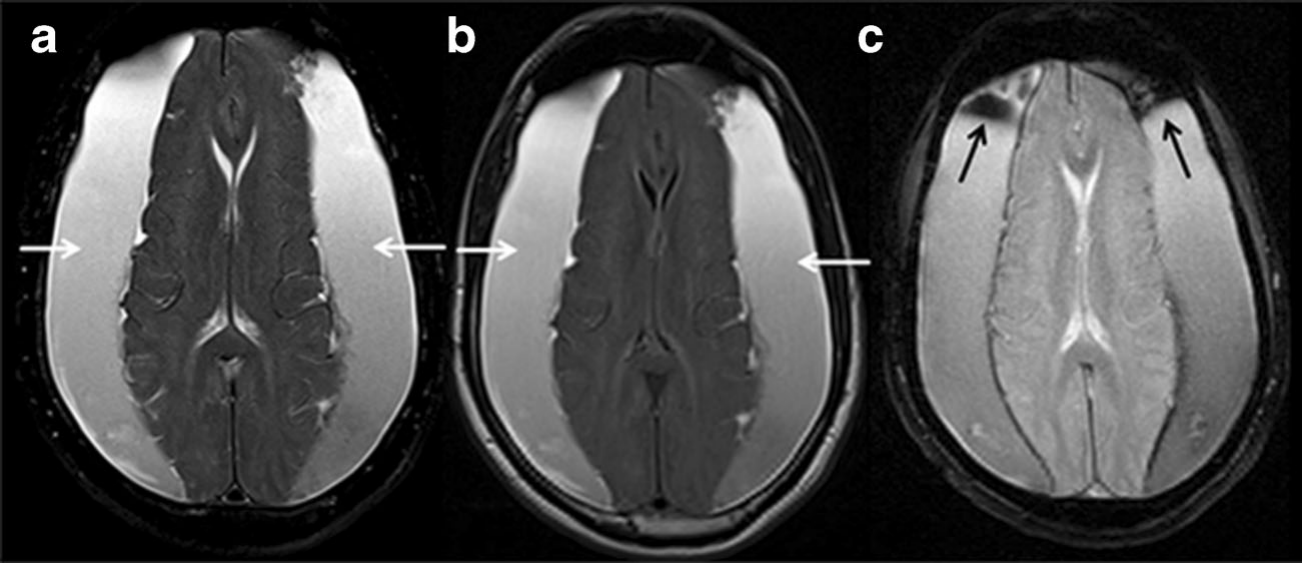
Chronic subdural hematoma (SDH). A 73-year-old male with trivial head trauma later developed progressive loss of cognitive function. **a & b** axial T2W and FLAIR sections of brain show hyperintense extra axial fluid collection in bilateral cerebral convexities (*white arrows*) with severe compression of adjacent cerebral hemispheres and effacement of the ventricles & sulci. **c** axial GRE image of the brain demonstrate predominantly hyperintense fluid in bilateral cerebral convexities with hypointense margin on the medial aspect. There are also localized hypointensities (blooming susceptibility artefacts from the blood products) on the anterior aspects of the collections (*black arrows*)

Chronic SDH is the most frequent cause of traumatic intracranial bleeding in the elderly population, especially in the background of cerebral atrophy. The mechanism of dementia can be explained by direct brain compression, reduction in whole brain cerebral blood flow (CBF) and generalized astrocyte reactivity. Approximately 45 % of patients with chronic SDH can have amnesia and cognitive deficits. This is considered to be one of the treatable forms of dementia and reversible upon surgical evacuation. On CT, chronic SDH often appears as a hypodense concavo-convex collection. On T1W and FLAIR MRI, it appears minimally hyperintense in comparison to CSF due to low paramagnetic methemoglobin and high protein content (Fig. 3). Membranes with loculations and varying stages of blood products should raise the suspicion of recurrent haemorrhage [11].

### Neurodegenerative

#### Dementia with Lewy bodies (DLB)

Dementia with Lewy bodies (DLB) is considered as the second most common type of degenerative dementia after Alzheimer disease (AD). Cognitive dysfunction is the frequent initial presentation of DLB, with dementia eventually occurring in all cases. Unlike Alzheimer disease (AD), the patients with DLB often present with early impairments in attention, executive and visuospatial function, with memory affected later in the course of the disease [12]. Other clinical manifestations are visual hallucinations, dysautonomia, parkinsonism, sleep disorders, and neuroleptic sensitivity. Radiologic features are not specific, but may be supportive in the diagnosis of DLB. Generalized brain atrophy may be noticed on MRI, similar to other neurodegenerative dementia. Few studies have shown atrophy of the putamen and dorsal mesopontine gray matter in DLB compared with AD on volumetric MRI scans [13]. As per one study, there can be reduced fractional anisotropy in the parieto-occipital white matter tracts on diffusion tensor imaging in DLB, but not in AD [14]. Single-photon emission computed tomography (SPECT) and positron emission tomography (PET) scans may show decreased perfusion and metabolism in the occipital areas [15]. The common differential diagnoses for DLB are Alzheimer disease (AD), Parkinson disease (PD), vascular dementia, other neurodegenerative dementias, and certain psychiatric disease. The diagnosis of DLB is primarily based on the clinical presentations, with radiologic features serving as helpful tool.

#### Dementia in Parkinson disease (PD)

Dementia is common and typically appears late in Parkinson disease (PD). Milder cognitive impairment represented by executive dysfunction and impaired visuospatial function is usually noted early in the disease [16]. Other associated clinical features are visual hallucinations, bradykinesia, rigidity and resting tremor. There are no specific imaging features for Parkinson disease with dementia; however, one of the recent neuroimaging studies has described the high accuracy of diagnosing PD by demonstrating absence of normal hyperintensity in the nigrosome-1 of the dorsolateral substantia nigra (Swallow Tail sign) on Susceptibility Weighted Imaging (SWI) MR sequence [17]. The most common differential diagnosis is Dementia with Lewy bodies (DLB). In PD, dementia generally occurs in the setting of well-established parkinsonism, whereas in DLB, dementia usually appears along with or before the development of parkinsonian signs. Quantitative morphometric MRI study can help in differentiating both entities with evidence of more pronounced cortical atrophy in DLB compared to PD. [18] Other differential diagnoses include progressive supranuclear palsy (PSP), corticobasal degeneration, Alzheimer disease and vascular dementia.

#### Multiple system atrophy- cerebellar type (MSA-C)

**Fig. 4:**
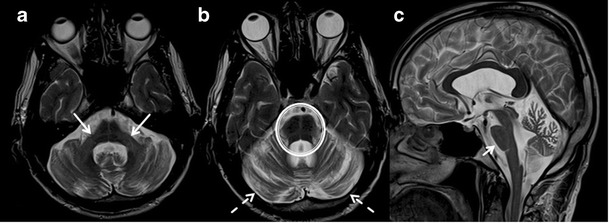
Multisystem atrophy-cerebellar variant (MSA-C). A 79-year-old female with a two-year history of urinary incontinence, ataxia, rigidity, generalized atrophy and cognitive decline. **a** axial T2 W section of the posterior fossa shows atrophy of bilateral brachium pontis with hyperintense signal changes (*white arrows*). **b** axial T2W image at the level of pons demonstrates typical hot cross bun sign (*white circle*) and significant atrophy and T2W hyperintensity in bilateral cerebellar hemispheres (*white dashed arrows*). **c** midline sagittal T2W image of brain is showing atrophy of pons mostly on the anterior aspect (*short white arrow*)

MSA-C was formerly called olivopontocerebellar degeneration, with onset at a mean age of 56 to 60 years. It is a sporadic disease with a combination of Parkinsonism symptoms, autonomic failure and cerebellar ataxia. Dementia is uncommon and of the frontal type, but is found in a minority of patients [19]. There is neuronal cell loss and gliosis in inferior olivary and pontine nuclei, cerebellar hemispheres and vermis. Common MR imaging features are selective atrophy of pons, middle cerebellar peduncles, cerebellar hemispheres and inferior olives (Fig. 4). An abnormal hyperintense signal that results from the degeneration of the transverse pontine fibres and pontine raphe gives the appearance of a “Hot Cross Bun” sign on T2W/FLAIR images (Fig. 4b) [20]. Although a non-specific finding, it has also been observed in variant Creutzfeldt-Jakob disease, spinocerebellar atrophy types 2 and 3 and in parkinsonism secondary to vasculitis.

#### Progressive supranuclear palsy (PSP)

**Fig. 5:**
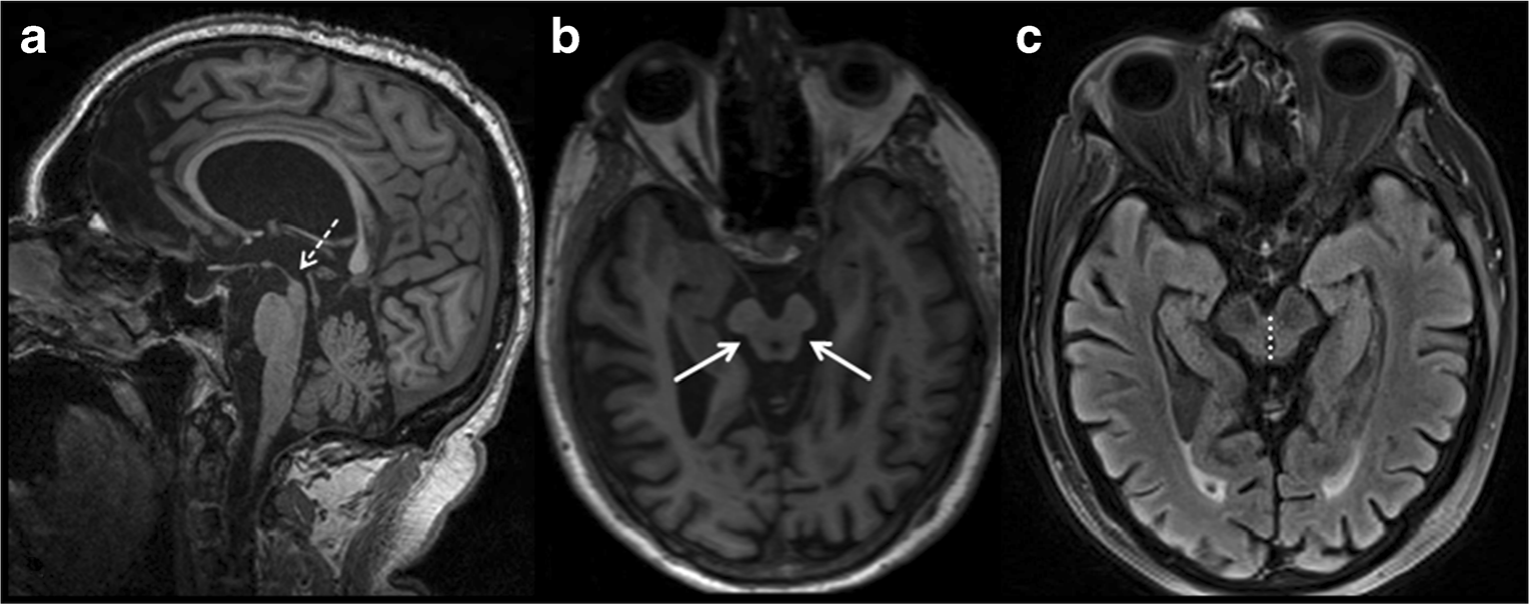
Progressive Supranuclear Palsy (PSP). A 79-year-old male with gait ataxia and frequent falls with forgetfulness and loss of interest in routine activity. **a** sagittal T1W image of the brain shows loss of superior convexity of midbrain: “Humming bird or penguin sign” (*white dashed arrow*). **b** axial T1W section of the brain at the level of midbrain reveals abnormal concavity of lateral margin of midbrain: “morning glory sign” (*white arrows*). **c** axial FLAIR imaging of brain is demonstrating decreased anteroposterior diameter of midbrain (*white dashed arrow*)

PSP is the most common form of atypical parkinsonian disorder, which can present with early executive dysfunction or subcortical dementia and clinically presents with supranuclear gaze palsy and motor symptoms. [21] Like FTLD, PSP is also related to abnormal tau protein deposition. Midbrain and superior cerebellar peduncle atrophy are classic neuroimaging features. Loss of normal superior midbrain tegmentum convexity with a relatively preserved pons on T1W mid-sagittal images gives the “hummingbird or penguin” beak appearance (Fig. 5a). Atrophy of superior cerebellar peduncles strongly correlates with disease duration. Reduced anteroposterior midbrain diameter at the level of superior colliculus gives the “Mickey Mouse” appearance on axial images (Fig. 5c) [22]. Axial images may also show abnormal concavity of the lateral tegmental margin, giving the impression of a “morning glory” flower (Fig. 5b) [23]. A number of studies have used quantitative measurements to establish the specificity of atrophy pattern noted in PSP, and tried to differentiate from idiopathic parkinson's disease or MSA-P [24]. The anterior-posterior diameter and average area of the midbrain were found to be significantly lower in patients with PSP (56 mm^2^) compared to idiopathic Parkinson disease (103 mm^2^) or multiple system atrophy-parkinsonism (MSA-P) (97 mm^2^) [25]. Similarly, PSP has shown a significantly higher ratio of the mid-sagittal pons to midbrain area compared with matched controls, multiple system atrophy or Parkinson disease. Few studies have suggested that magnetic resonance-parkinsonism index (MRPI) can be used to distinguish PSP from Parkinson disease and atypical parkinsonian syndrome [26]. It is an index calculated from the formula (P/M) × (MCP/SCP), which requires measurement of the area of the pons (P) and midbrain (M) and width of the middle cerebellar peduncle (MCP) on sagittal T1-weighted MRI and the width of the superior cerebellar peduncle (SCP) on coronal MRI.

#### Frontotemporal lobar dementia (FTLD)

**Fig. 6:**
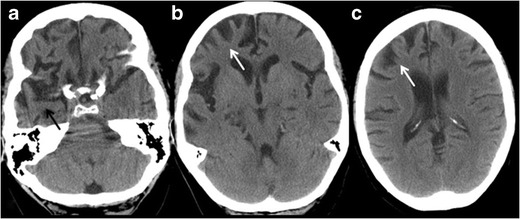
Behavioural variant Frontotemporal dementia (bvFTD). A 53-year-old male with progressive personality change and behavioural problems with cognitive decline. **a**, **b & c** axial CT sections of brain show severe right frontal lobe atrophy (*white arrow*), as well as some atrophy of right anterior temporal lobe (*black arrow*)

FTLD is a group of neurodegenerative diseases with a relatively rarer form of rapidly progressive dementia, characteristically involving the fronto-temporal lobes and insula. Clinically it comprises three subtypes: behavioral variant frontotemporal dementia (bvFTD), semantic dementia (SD) and nonfluent aphasia. The bvFTD is characterized by marked personality changes and behavioural problems, the most aggressive subtype with earliest average age of onset (fifth to sixth decade) and male preponderance [27]. SD or the temporal variant is also known as the least aggressive variant, and typically presents with either word finding difficulty or prosopagnosia (inability to recognize faces), depending on the predominant temporal lobe predilection [28]. Patients with non-fluent aphasia generally present with speech apraxia [29]. The characteristic neuropathological finding in FTLD is tau proteinopathy, as in AD. Neuroimaging is non-specific in the early phase of disease with overlap among subtypes, and is used primarily to exclude other forms of dementia. With clinical progression, FTLD subtypes frequently show specific patterns of focal cortical atrophy with the classic “knife edge” appearance involving frontal and/or temporal lobes. In the bvFTD subtype, bifrontal atrophy is the most common finding, which usually progresses to involve the temporal lobes at a later stage of the disease [30]. Behavioural changes are usually attributed to predominant involvement of the right frontal lobe (Fig. 6). The SD variant has predominant anterior temporal lobe atrophy and is more frequently marked on the left side, unlike AD, in which the temporal lobe involvement tends to be symmetric with no antero-posterior gradient. Prominent atrophy of the left perisylvian region is characteristic of non-fluent aphasia [31]. Additionally, molecular imaging with either FDG-PET or rCBF (relative Cerebral Blood Flow) SPECT and quantitative analysis using 3D T1 MRI have shown good specificity in differentiating FTLD and AD based on identification of asymmetric and predominantly anterior versus posterior pattern of hypometabolism/perfusion and atrophy.

#### Huntington’s disease (HD)

**Fig. 7:**
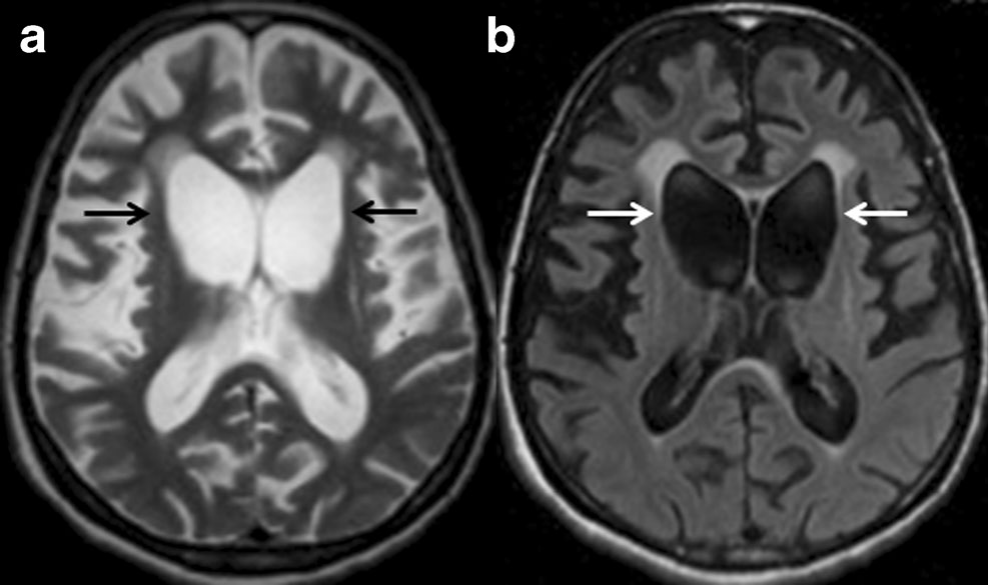
Huntington’s disease. A 62-year-old male presented with abnormal choreiform movement of the limbs, psychiatric manifestations and dementia. **a** axial T2W image at the level of lateral ventricles is showing significant atrophy of bilateral caudate nuclei (*black arrows*) and frontal lobes. **b** corresponding axial FLAIR image also demonstrates significant caudate atrophy (*white arrows*) and enlargement of frontal horns

HD is an autosomal dominant, inherited progressive neurodegenerative disorder caused by a trinucleotide (CAG) repeat expansion in the huntingtin gene. It is characterized by abnormal choreiform movements, psychiatric symptoms and early onset dementia [32]. Cognitive deficits are inevitable in HD and common clinical manifestations are executive dysfunction, lack of insight and slowed processing. Caudate atrophy is the typical neuroimaging finding on axial MRI images (Fig. 7), and changes in linear caudate measurements correlate with severity of cognitive dysfunction [33]. Abnormal metabolic changes can also be noted on positron-emission tomography (PET) and functional MRI [34]. The differential diagnosis of HD is broad and ranges from benign familial chorea to hereditary and acquired neurodegenerative diseases. The diagnosis of HD is principally based upon a combination of clinical features, family history and presence of trinucleotide (CAG) repeat expansion in the HTT gene.

### Inflammatory/Infective

#### Creutzfeldt-Jakob disease (CJD)

**Fig. 8:**
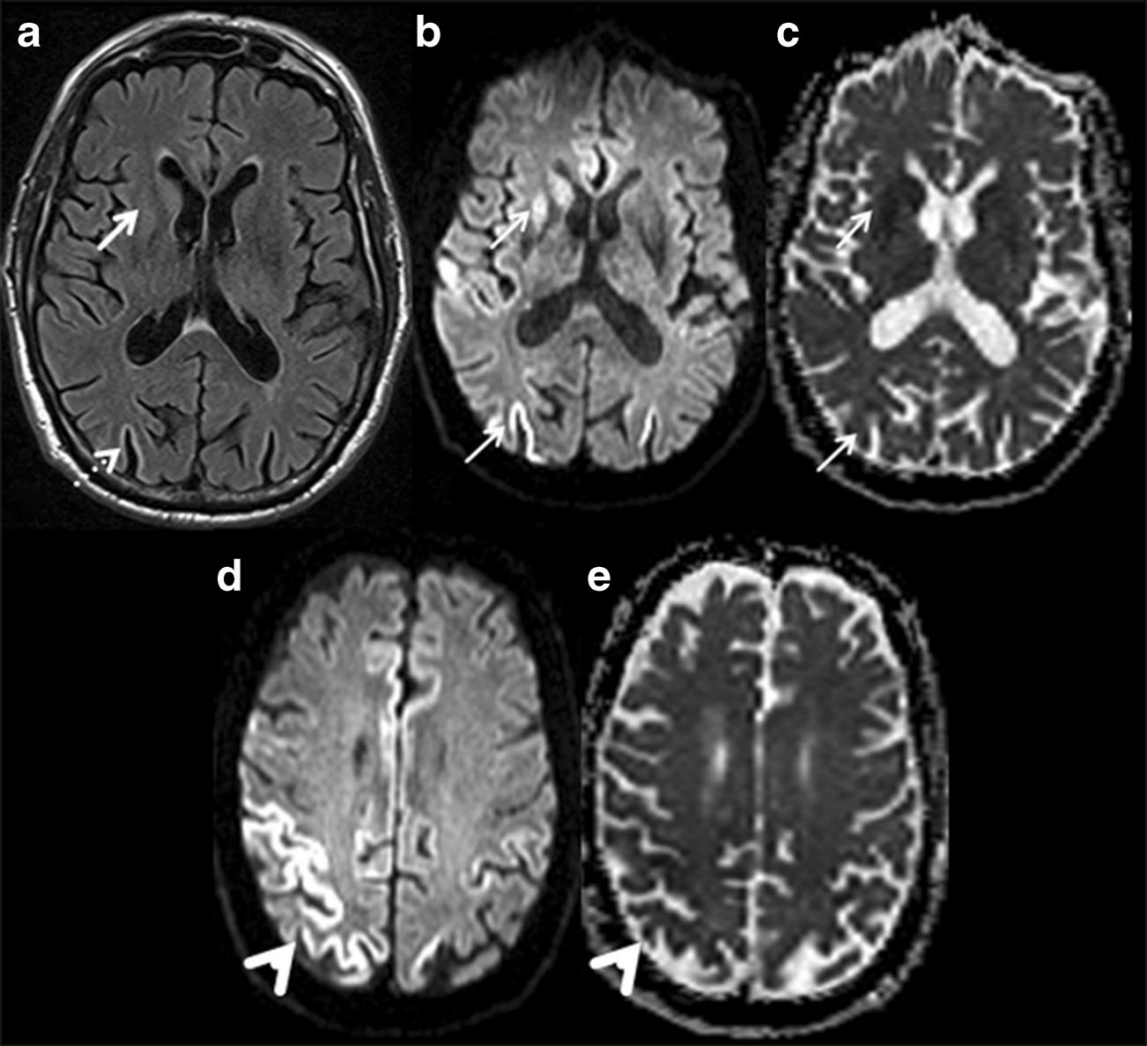
Creutzfeldt-Jakob Disease (CJD). **a** FLAIR axial image of the brain shows hyperintensity in the right caudate head, putamen (*white arrow*) and occipital cortex (*white dashed arrow*). **b & c** diffusion weighted image and ADC map reveal restriction of diffusion in the right caudate head, putamen and occipital cortex. **d & e** diffusion weighted image and ADC map at higher level demonstrate restriction of diffusion in the right frontoparietal cortex

CJD is a fatal prion disease causing rapidly progressive dementia due to sub-acute spongiform encephalopathy. It commonly presents in the fifth to seventh decade of life with a triad of subacute dementia, myoclonus and characteristic triphasic EEG pattern. Elevated tau and/or 14-3-3 protein is seen on CSF analysis. MRI is the most suitable neuroimaging technique in the diagnosis of CJD, with T2W, FLAIR and DWI sequences being the most essential sequences of the MRI protocol. The key MRI features in CJD are T2W/FLAIR hyperintensity in the caudate, putamen, anterior cingulate gyrus and thalamus. Widespread involvement of the cerebral cortex is also a characteristic feature [35]. DWI is very sensitive and shows diffusion restriction, especially in early stage of the disease, with no visible changes on T2W/FLAIR sequences (Fig. 8) [36]. Generalized atrophy is usually noted in late or terminal CJD. Variant CJD (vCJD) commonly presents with psychiatric symptoms and selective T2W/FLAIR hyperintensity with diffusion restriction in the medial and dorsal thalami (pulvinar), giving the appearance of “hockey-stick” or “pulvinar sign” [37]. A combination of clinical, neuroimaging and neuropathological findings frequently leads to the definitive diagnosis of CJD.

#### HIV-Associated dementia (HAD)

**Fig. 9:**
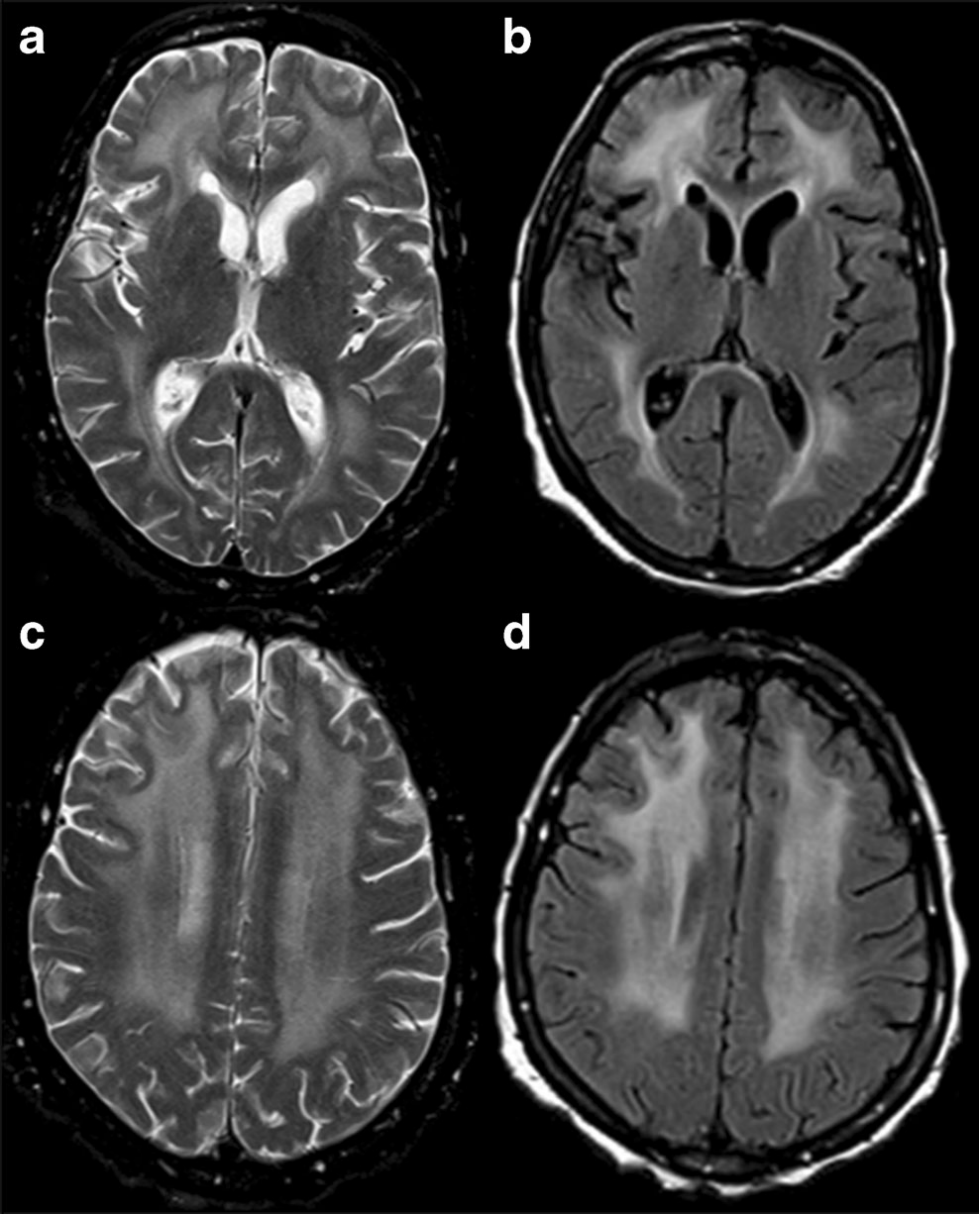
HIV-Associated Dementia (HAD). A 56-year-old female with a several-month history of generalized weakness, confusion and memory loss. **a & b** axial T2W and FLAIR images of the brain at the level of ventricles show diffuse symmetrical white matter hyperintensity and mild generalized atrophy. **c & d** axial T2W and FLAIR images at the level of centrum semiovale reveal similar diffuse symmetrical hyperintensity in the white matter

Human Immunodeficiency Virus (HIV-1) has created a global crisis, with approximately 50 million people infected worldwide. HAD has also been previously referred to as AIDS dementia complex, HIV encephalitis or encephalopathy, and is seen in approximately 25–52 % of patients with HIV infection, depending on disease state and anti-retroviral therapy. Patients develop insidious onset intellectual disturbance, psychomotor slowing and memory impairment. Generalized brain atrophy is the most common imaging finding. MRI may show patchy T2W/FLAIR hyperintensities in periventricular and subcortical white matter, which tends to be more diffuse and confluent with disease progression (Fig. 9) [38]. Lack of brain parenchymal or meningeal enhancement differentiates it from other opportunistic infections. Bilateral symmetrical hyperintensity in basal ganglia, thalami and brainstem has been described in the literature [39]. Advanced imaging techniques like magnetization transfer (MT) and proton MR spectroscopy (MRS) have shown low MT and decreased glutamate, NAA/choline ratios with increased myoinositol in the affected brain regions [39].

#### Sjogren’s syndrome (SS)

**Fig. 10:**
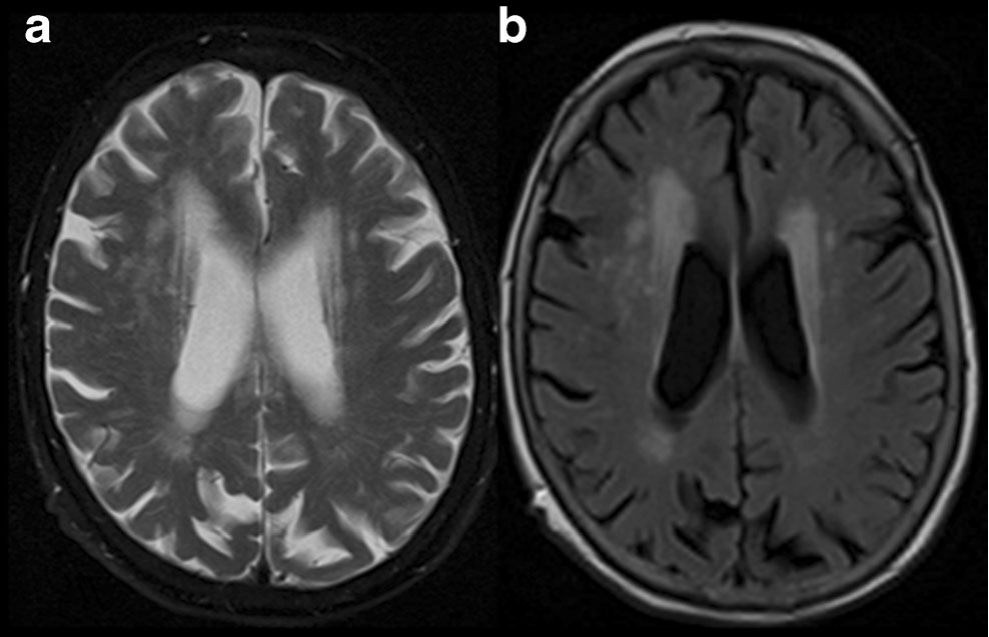
Sjogren’s syndrome. A 71-year-old female presented to hospital with unsteadiness, frequent falls and dementia with features of Sjogren’s syndrome. **a & b** axial T2W and FLAIR images of the brain show nonspecific patchy confluent hyperintensities in bilateral periventricular white matter

Sjogren’s syndrome is one of the most common autoimmune diseases, affecting the exocrine glands and manifesting as sicca syndrome. CNS involvement is noted in approximately 20 % of patients, presenting with transverse myelitis and aseptic meningitis. It can manifest as steroid-responsive treatable dementia, which is caused by angiitis or nonvasculitic autoimmune inflammatory meningoencephalitis (NAIM). Cognitive and behavioural disorders like forgetfulness and bradyphrenia are some known presentations. MR imaging can often be normal or show non-specific perivascular and subcortical small punctate white matter lesions (Fig. 10) [40, 41].

#### Multiple sclerosis (MS)

**Fig. 11:**
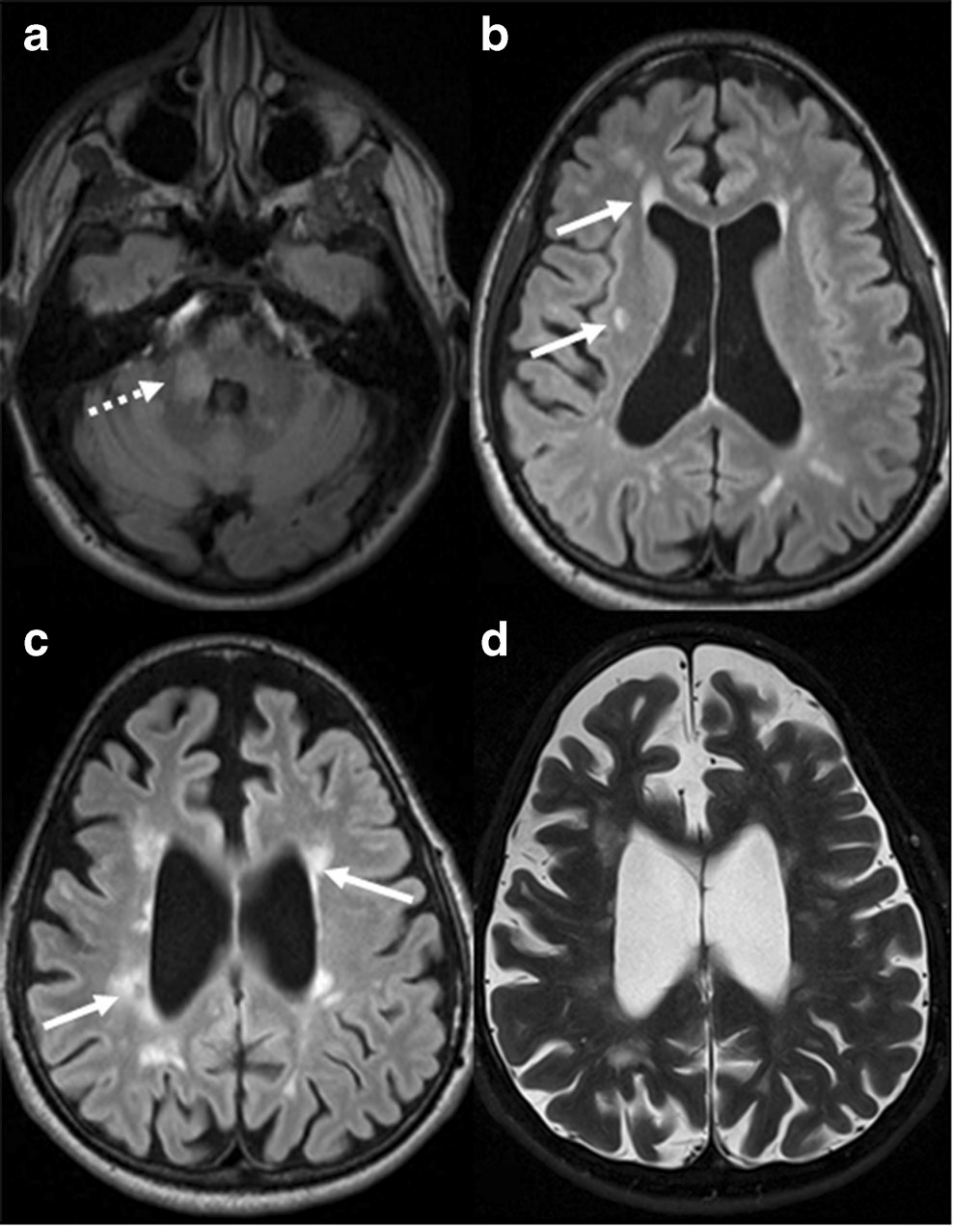
Mutliple Sclerosis (MS). A 40-year-old female with history of relapsing remitting multiple sclerosis (RRMS) for the last 5 years, recently developed progressive memory decline. **a** axial FLAIR image of the posterior fossa shows a hyperintense demyelinating lesion in the right brachium pontis (*white dashed arrow*). **b**, **c & d** axial FLAIR and T2W images at the level of lateral ventricles demonstrate multiple hyperintense lesions in periventricular white matter (*white arrows*) and generalized atrophy

MS is a demyelinating disease involving both white and gray matter. Focal neurological deficits caused by lesions in an eloquent long tract, like optic neuritis or a spinal cord syndrome, are common presentations. Nearly one in 20 patients can manifest with dementia and the common imaging finding is nonspecific generalized atrophy (Fig. 11) [42]. The degree of cognitive decline is proportional to the disease burden on MRI. Abnormal Magnetization Transfer Ratio (MTR) or Fractional Anisotropy (FA) have been reported in the cortex of MS patients with reduced NAA and increased mI/Cr (mI- myo inositol, Cr- Creatine) ratios in deep grey matter on MRS, suggesting both axonal injury and gliosis [42].

### Metabolic/Genetic

#### Delayed post hypoxic leukoencephalopathy (DPHL)

**Fig. 12:**
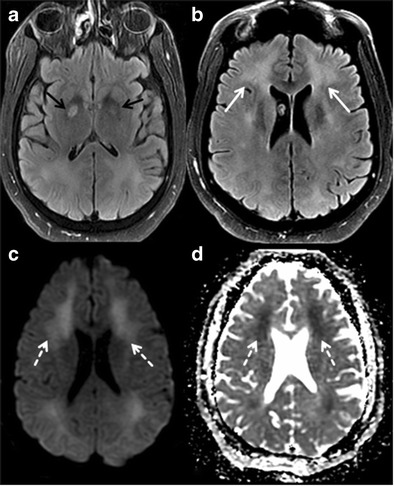
Delayed Post Hypoxic Leukoencephalopathy (DPHL). A 42-year-old male prisoner with history of substance abuse presented with altered level of consciousness, confusion, ataxia and memory loss. **a** axial FLAIR image is showing symmetrical necrosis of bilateral globus pallidus (*black arrows*). **b** axial FLAIR image at the level of ventricles demonstrates symmetrical periventricular and deep white matter hyperintensity (*white arrow*). **c & d** diffusion weighted image and ADC map reveal symmetrical restriction of diffusion in the deep white matter

There are three basic mechanisms of developing hypoxic-ischemic brain injury- hypovolemia (cardiac failure), hypo-oxygenation (respiratory failure) and impaired oxygenation (CO poisoning) [43]. Initial clinical presentation includes a brief period of coma or altered consciousness. Recovery from the initial insult can manifest with short-term memory loss, confusion, ataxia and poor performance in executive function. Delayed post-hypoxic demyelination is considered to be the cause for cognitive deficits [44]. Diffuse cortical, periventricular white matter and deep gray matter T2W/FLAIR hyperintensity with DWI restriction are frequently noted on MRI (Fig. 12) [44]. The imaging appearance may overlap in few other conditions such as PRESS, spongiform leukoencephalopathy secondary to inhaled heroin and metachromatic leukodystrophy; however, history of hypoxia and cognitive decline are helpful in differentiating DPHL from other conditions.

#### Acute methanol toxicity

**Fig. 13:**
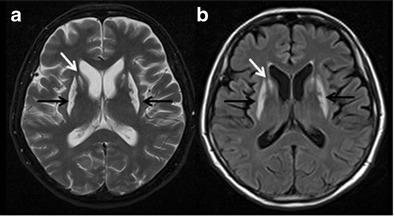
Methanol toxicity. A 52-year-old female with a history of accidental methanol ingestion, presented with cognitive decline and rigidity in the extremities. **a & b** axial T2W and FLAIR images of the brain show necrosis of bilateral putamen (*black arrows*) and head of caudate nuclei (*white arrow*)

Methanol is a highly toxic, colourless liquid that resembles ethanol in taste and smell. Intoxication is common after suicidal or accidental ingestion and fraudulent adulteration of alcoholic beverages. It results in severe metabolic acidosis and optic necrosis or demyelination leading to vision loss. Severe toxicity may progress to Parkinson-like extrapyramidal syndrome, cognitive deficits/dementia, permanent neurological deficits and death [45]. Both CT and MRI are helpful in demonstrating the changes in the brain. Bilateral putaminal necrosis with varying degrees of haemorrhage is the crucial imaging feature (Fig. 13). Cerebral and intraventricular haemorrhage, cerebellar and subcortical white matter necrosis and diffuse cerebral oedema are other common imaging manifestations [46]. Imaging differential diagnosis could include carbon monoxide poisoning, stroke, Wilson and Leigh disease.

#### Central pontine myelinolysis

**Fig. 14:**
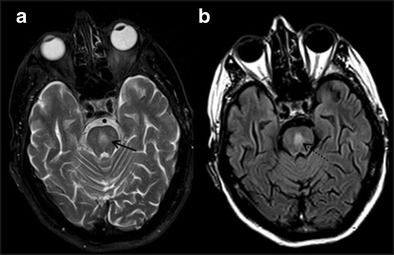
Central Pontine Myelinolysis (CPM). A 61-year-old male with history of ETOH abuse with decrease in function, poor balance and dementia. **a & b** axial T2W and FLAIR images at the level of pons show central pontine hyperintensity (*black arrow*)

Central pontine myelinolysis is an acute demyelinating process affecting the central pontine fibres and frequently associated with chronic alcoholism, nutritional deficiency and rapid correction of hyponatremia (osmotic demyelination). Spastic quadriparesis, pseudobulbar palsy, altered level of consciousness and coma are usual presentations. Dementia component and behavioural changes are likely from callosal involvement [47]. Symmetric ‘Trident shaped’ T2W/FLAIR hyperintensity in the central pons with sparing of corticospinal tract are characteristic MR imaging features (Fig. 14). Diffusion restriction may be seen in the early phase with occasional peripheral post contrast enhancement [48]. Stroke, MS, encephalitis, glioma and toxic encephalopathy are common MR imaging differential diagnoses.

#### Fragile X-associated tremor/Ataxia syndrome (FXTAS)

**Fig. 15:**
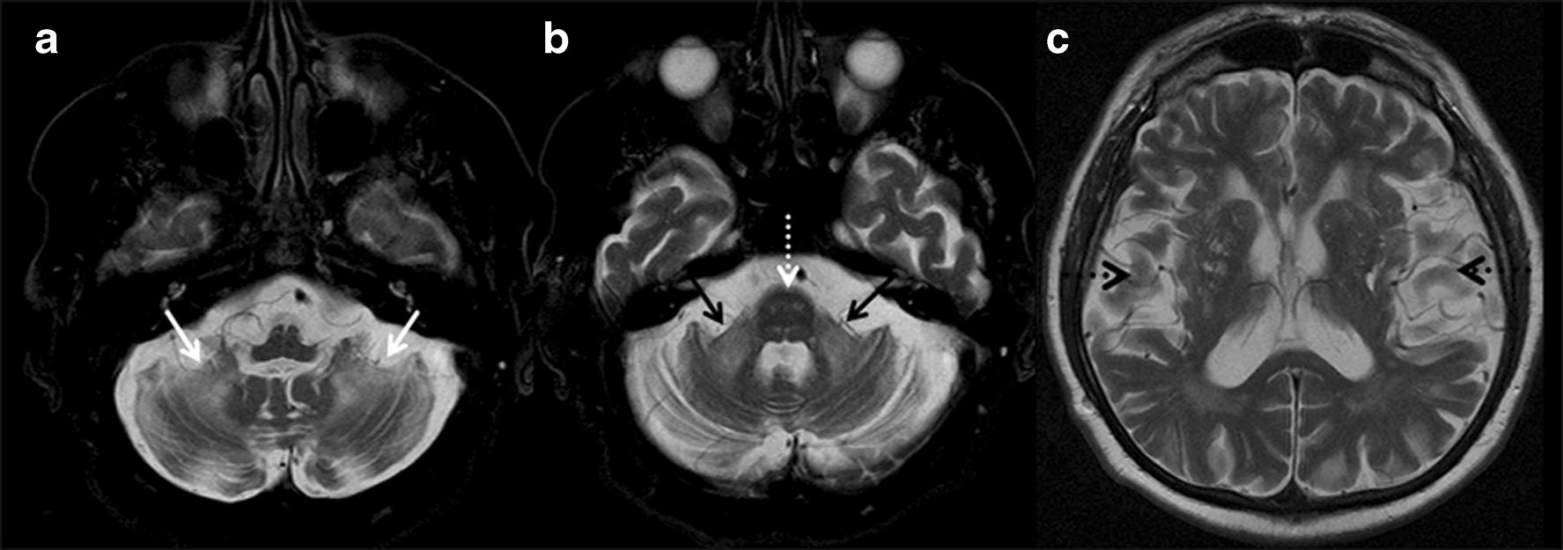
Fragile X- Associated Tremor/Ataxia Syndrome (FXTAS). A 61-year-old male presented with a two-year history of tremor, rigidity in all four limbs, paresthesia with recent memory decline. **a** axial T2W image of the posterior fossa show atrophy and T2W hyperintensity in bilateral cerebellar hemisphere (*white arrows*). **b** axial T2W section of the brain at the level of pons demonstrate symmetrical hyperintensity in pons (dashed white arrow) and hyperintensity in bilateral middle cerebellar peduncles (black arrows). **c** axial T2W image of the brain at the level of lateral ventricles is revealing significant atrophy and T2W hyperintensities in bilateral cerebral hemispheres

FXTAS is a late onset X linked genetic cause of mental retardation and autism due to trinucleotide repeat mutation of the Fragile X Mental Retardation 1 (FMR1) gene, and is commonly seen in males between the ages of 50 and80 years. The clinical presentation includes intentional tremor, ataxia, neuropathy, and personality changes in association with memory loss. Atrophy of the cerebral and cerebellar hemispheres, middle cerebellar peduncles, pons, medulla and corpus callosum are the important imaging features. Symmetric T2W/FLAIR hyperintense lesions in the cerebellar hemispheres close to the dentate nucleus, pons and bilateral middle cerebellar peduncles (MCP sign) are commonly noted (Fig. 15a & b). In addition, symmetric patchy and/or confluent hyperintensities can be seen in periventricular and deep white matter of cerebral hemispheres with sparing of subcortical U fibers, cortical and deep gray matter and corpus callosum (Fig. 15c) [49].

### Miscellaneous

#### Intracranial dural AV fistula (dAVF)

**Fig. 16:**
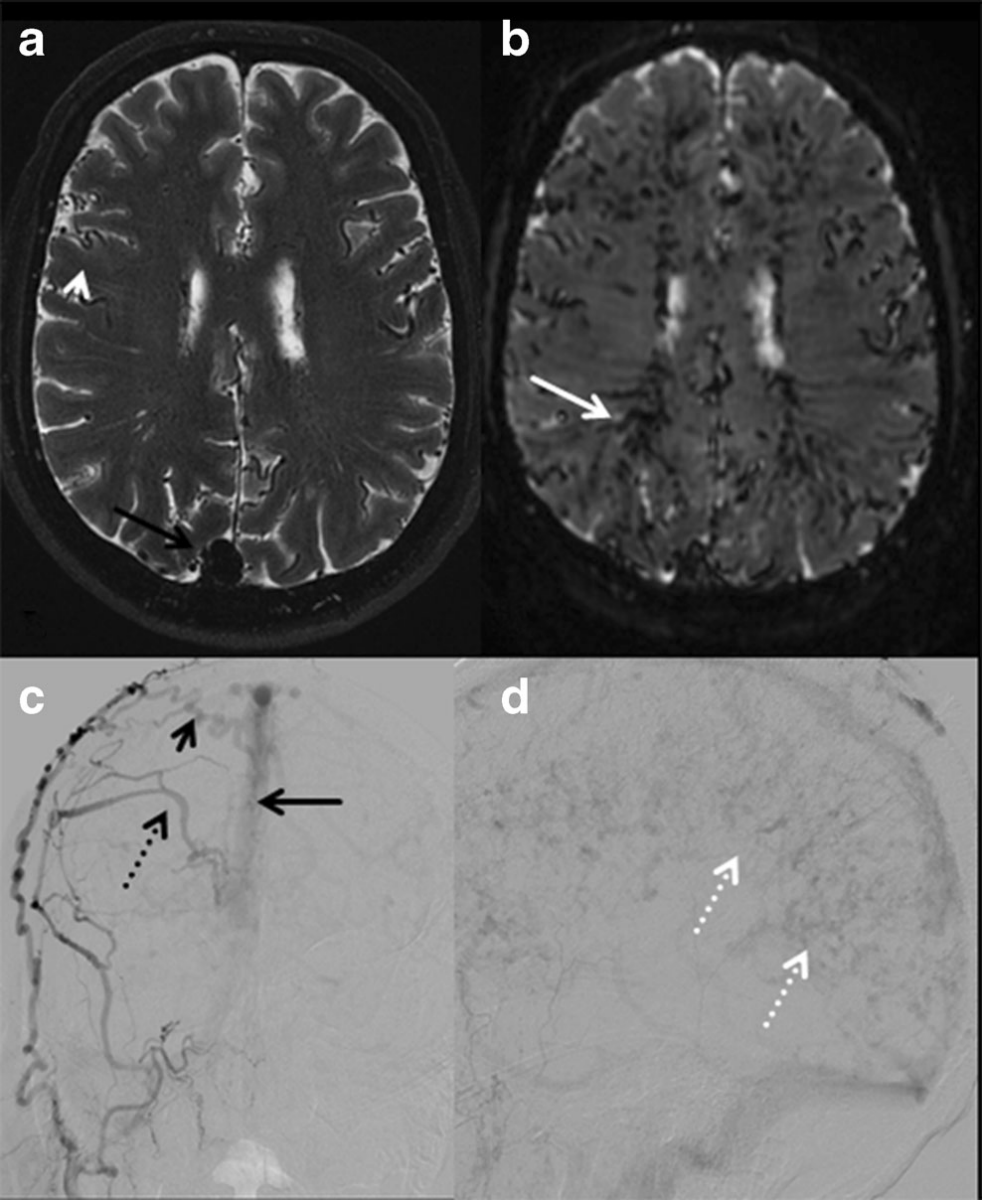
Intracranial dural AV fistula. A 69-year-old male with a 1-month history of progressive cognitive decline. **a & b** axial T2W and GRE images of the brain show prominent superior sagittal sinus (*black arrow*) with multiple tortuous superficial cortical (*arrow head*) and medullary veins (*white arrow*). **c** right external carotid artery injection reveals dural AV fistula with abnormal communication between superior sagittal sinus (*black arrow*) and arterial feeders from right middle meningeal artery (*dashed black arrow*) and transosseous branches of right superficial temporal artery (*short black arrow*). **d** Late venous phase of the catheter angiogram shows prominent medullary venous drainage giving the appearance of pseudophlebitic appearance (*dashed white arrows*)

dAVF is an acquired pathological shunt between intracranial arteries and dural venous sinuses, meningeal or cortical veins. It mostly presents in the fifth or sixth decades and the common locations are in transverse, sigmoid and cavernous sinuses [50]. Many dAVFs remain asymptomatic or have a benign course, but can present with cranial neuropathy, seizures, parkinsonism, cerebellar symptoms and intracranial haemorrhage in higher grade dAVFs. Cognitive decline and global dementia are uncommon clinical manifestations most likely resulting from venous hypertension and brain ischemia associated with impaired venous drainage [51]. Unenhanced CT is particularly limited to detecting intracranial haemorrhage and brain oedema. MRI is mainly suitable for demonstrating ectatic vessels, venous pouches, and signs of venous congestion such as white matter signal changes, haemorrhage, or venous infarcts (Fig. 16a and b). Dynamic CTA and time resolved MRA can be useful for demonstrating the dAVF and its relation to surrounding brain and skull, and thus may help in treatment planning. Digital subtraction angiogram is most accurate in demonstrating cranial dAVF, with superior visualization of arterial feeders, site of AV shunting and venous drainage (Fig. 16c & d) [51]. Although a very rare cause of dementia, it can be reversible upon successful embolization of the fistula.

#### Normal pressure hydrocephalus (NPH)

**Fig. 17:**
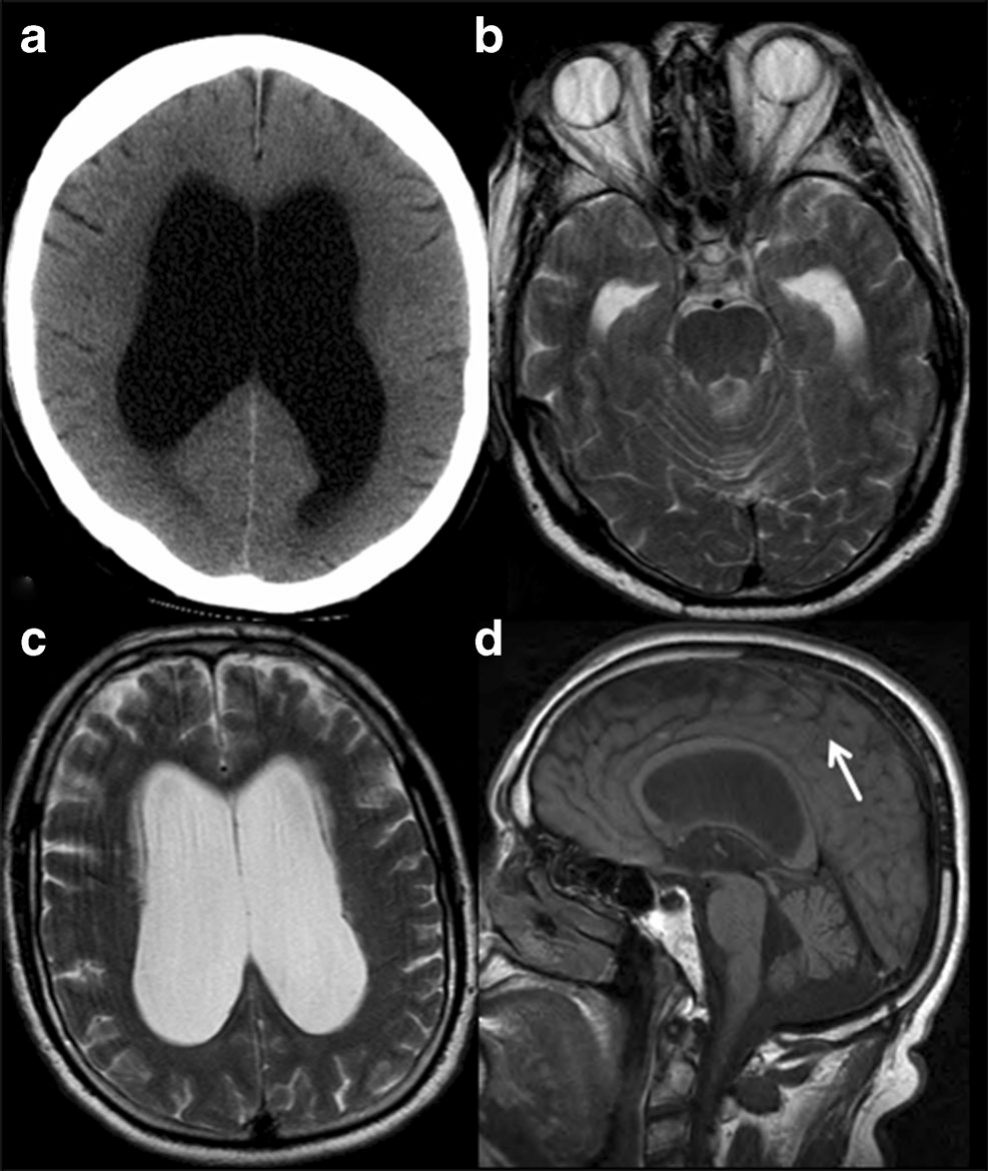
Normal Pressure Hydrocephalus (NPH). A 75 -ear-old male presented with progressive gait ataxia and memory loss. **a** axial CT section of the brain shows dilated lateral ventricles with effaced sulci. **b & c** axial T2W images of brain at the level of ventricles demonstrate dilatation of all the ventricles with prominent CSF flow void in the fourth ventricle (*black arrow*). **d** paramedian sagittal T1W section reveals ballooned corpus callosum with effacement of sulci towards vertex (*white arrow*)

Normal pressure hydrocephalus represents ventricular dilatation and clinical triad of cognitive decline, gait disturbance and urinary incontinence with normal CSF opening pressure. Management of this condition is a dilemma because of diagnostic uncertainty, and requires careful patient selection for CSF shunting. The aetiology is still debatable and explained by two existing theories: 1) Impaired CSF absorption due to prior meningitis or subarachnoid haemorrhage and 2) Decreased white matter tensile strength due to ischemic changes [52]. Imaging plays a minor role and MRI findings that may suggest NPH are 1) disproportionate ventricular dilatation in comparison to sulcal prominence; 2) periventricular halo suggesting transependymal CSF flow; and 3) prominent CSF flow void at the aqueduct of Sylvius, attributable to excessive rapidly pulsatile CSF flow [53]. Ballooning of the corpus callosum is another imaging feature (Fig. 17). MRI cine flow velocity mapping measures the aqueductal CSF flow velocity and stroke volume is used at some centres to predict the response to shunt surgery.

#### Radiation necrosis

**Fig. 18:**
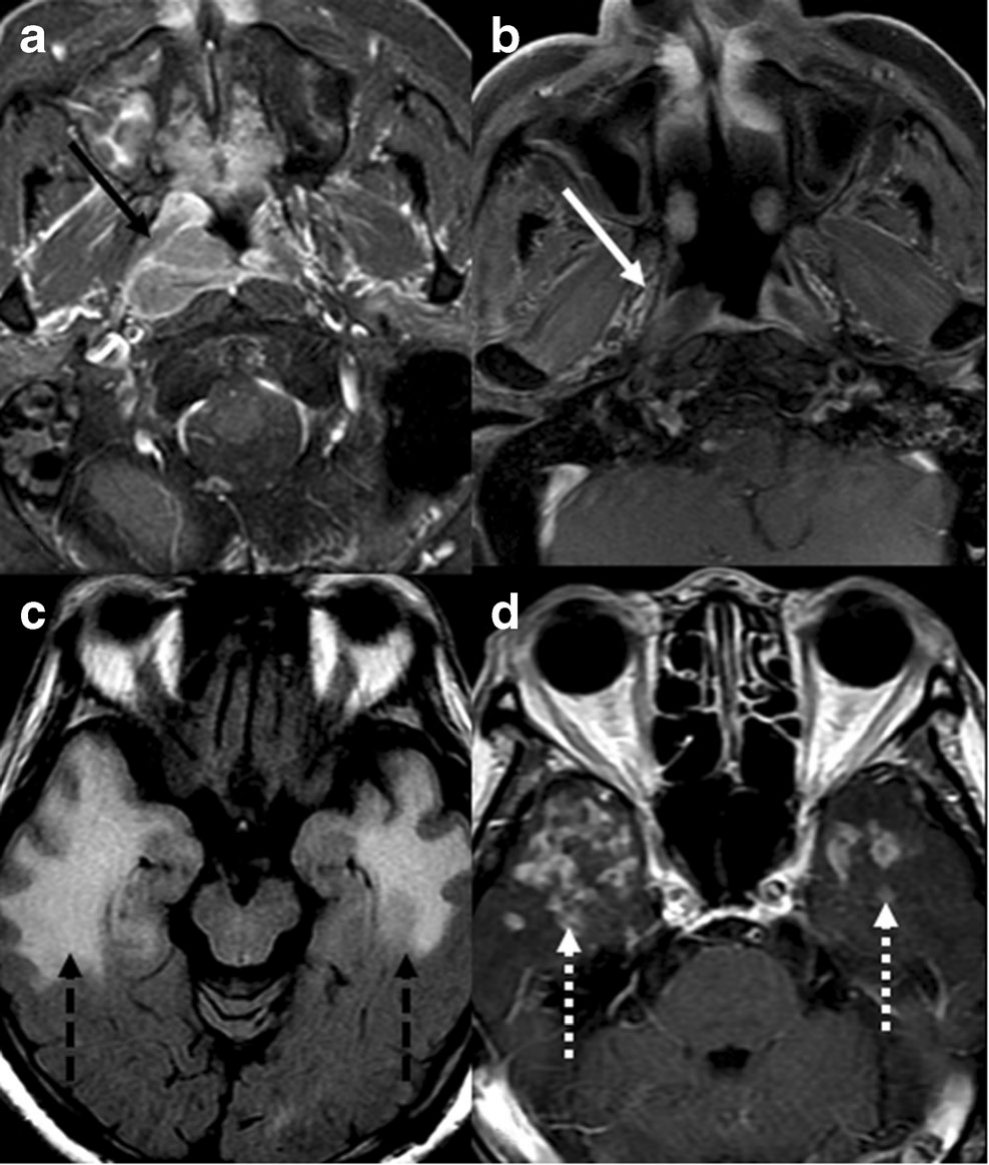
Radiation necrosis with dementia. A 52-year-old male with history of nasopharyngeal carcinoma with radiotherapy developed progressive decline in cognition after 7 years of treatment. **a** axial post contrast T1W image at the level of skull base (2003) shows heterogeneously enhancing right side nasopharyngeal carcinoma (*black arrow*). **b** axial post contrast T1W image of the skull base (2007) reveals complete resolution of the mass lesion (*white arrow*). **c & d** axial FLAIR and post contrast T1W images of the brain at the level of middle cranial fossa (2010) demonstrate extensive white matter hyperintensity (*black arrows*) and heterogeneous enhancement—“Swiss-cheese” pattern in bilateral temporal lobes (*white arrows*)

Patients irradiated for nasopharyngeal and oropharyngeal tumours can also present with cognitive and memory impairment due to bilateral temporal lobe involvement [54]. Radiation necrosis is a late complication occurring between 6 months to more than 10 years after radiotherapy. Diffuse T2/FLAIR hyperintense signal reflecting white matter oedema, cortical thinning and nodular, curvilinear or ring type post contrast enhancement are the common MRI features. The typical radiation necrosis enhancement has been described as a “Swiss-cheese” pattern (Fig. 18) [55]. MR perfusion and SPECT/PET studies may be useful in distinguishing viable tumour and radiation necrosis with low perfusion/hypometabolism in the latter [55].

#### Brain tumours

**Fig. 19:**
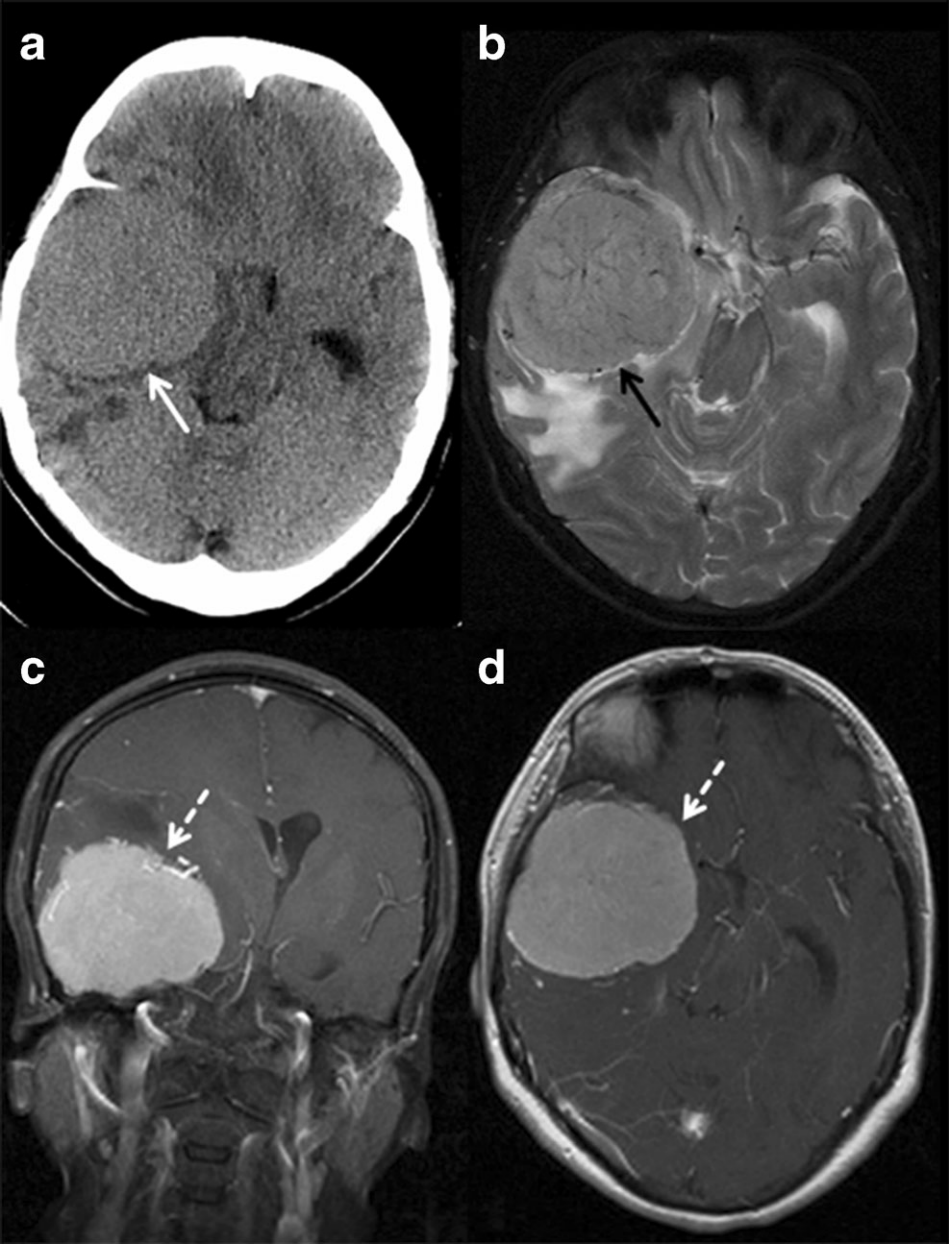
Right middle cranial fossa meningioma. A 55-year-old female presented with LOC and memory disturbance. **a** axial noncontrast CT section of the brain shows large isodense mass lesion (*white arrow*) in the right middle cranial fossa with significant mass effect. **b** axial T2W image of the brain at the level of middle cranial fossa shows large extraaxial mass lesion (*black arrow*) in the right middle cranial fossa with perilesional oedema and compression of adjacent right temporal lobe. **c & d** the lesion is showing homogeneous enhancement (*white dashed arrow*) on post contrast study

Both extra and intra axial tumours, when situated near eloquent areas, may present with slowly progressing neuropsychological, behavioural disturbance and dementia. It is potentially a reversible cause of dementia if amenable to surgical resection. Common locations are being middle cranial fossa, basal frontal regions, thalamus, corpus callosum, cingulate gyrus and limbic system (Fig. 19). Neuroimaging aids in excluding tumoral pathologies in unexplained behavioural and cognitive disturbances [56].

## Conclusion

Non-Alzheimer’s dementia comprises several pathologies varying from rapid onset to gradually progressive disease spectrum. Often there is considerable clinical overlap, and neuroimaging features tend to be non-specific during the initial stage of disease. The rationale of conventional neuroimaging is not to offer definitive diagnoses, but to suggest possible differential diagnoses and track evolving changes on follow-up studies. Newer molecular imaging bio-markers combined with conventional neuroimaging techniques hold promise in assessing applications of novel disease-modifying drugs for treatment and response evaluation. An important aspect of neuroimaging is also to recognize reversible or treatable causes of dementia that can potentially be treated or halt further disease progression.
